# LKB1 depletion-mediated epithelial–mesenchymal transition induces fibroblast activation in lung fibrosis

**DOI:** 10.1016/j.gendis.2023.06.034

**Published:** 2024-05

**Authors:** Zijian Xu, Elizabeth R. Davies, Liudi Yao, Yilu Zhou, Juanjuan Li, Aiman Alzetani, Ben G. Marshall, David Hancock, Tim Wallis, Julian Downward, Rob M. Ewing, Donna E. Davies, Mark G. Jones, Yihua Wang

**Affiliations:** 1 Biological Sciences, Faculty of Environmental and Life Sciences, University of Southampton, Southampton SO17 1BJ, UK; 2 Clinical and Experimental Sciences, Faculty of Medicine, University of Southampton, Southampton SO16 6YD, UK; 3 Institute for Life Sciences, University of Southampton, Southampton SO17 1BJ, UK; 4 NIHR Southampton Biomedical Research Centre, University Hospital Southampton, Southampton SO16 6YD, UK; 5 University Hospital Southampton, Southampton SO16 6YD, UK; 6 Oncogene Biology, The Francis Crick Institute, London NW1 1AT, UK

## Abstract

The factors that determine fibrosis progression or normal tissue repair are largely unknown. We previously demonstrated that autophagy inhibition-mediated epithelial-mesenchymal transition (EMT) in human alveolar epithelial type II (ATII) cells augments local myofibroblast differentiation in pulmonary fibrosis by paracrine signalling. Here, we report that liver kinase B1 (LKB1) inactivation in ATII cells inhibits autophagy and induces EMT as a consequence. In IPF lungs, this is caused by downregulation of *CAB39L*, a key subunit within the LKB1 complex. 3D co-cultures of ATII cells and MRC5 lung fibroblasts coupled with RNA sequencing (RNA-seq) confirmed that paracrine signalling between LKB1-depleted ATII cells and fibroblasts augmented myofibroblast differentiation. Together these data suggest that reduced autophagy caused by LKB1 inhibition can induce EMT in ATII cells and contribute to fibrosis via aberrant epithelial–fibroblast crosstalk.

## Introduction

Idiopathic pulmonary fibrosis (IPF) is a chronic, progressive, fibrotic lung disease of unknown aetiology ^1^. In IPF the current paradigm of disease pathogenesis proposes that the delicate alveolar architecture of the lung is disrupted by extracellular matrix (ECM) deposition as a consequence of repetitive micro-injuries to the alveolar epithelium, resulting in tissue scarring, increased stiffness and impaired gas exchange. Two anti-fibrotic drugs, nintedanib and pirfenidone, are approved worldwide for the treatment of IPF however whilst there is evidence that they can slow disease progression they cannot stop or reverse it ^2^ and so better treatments are urgently required.

We previously identified that alveolar epithelial type II (ATII) cells undergoing epithelial-mesenchymal transition (EMT) promote a pro-fibrotic microenvironment through paracrine signalling activating local fibroblasts ^3, 4, 5^. EMT is a dynamic, reversible process which has been implicated in embryonic development, wound healing, cancer metastasis and fibrosis ^6^. Induction of EMT in fibrosis has been linked to a variety of processes including autophagy inhibition which triggers EMT via the p62/*SQSMT1*-NFκB-Snail2 signalling pathway ^3, 7^ Autophagy (macro-autophagy) is a regulated self-management mechanism allowing the bulk or selective degradation of intracellular components and has been widely associated with several ageing processes including neurodegeneration, cancer and fibrosis ^8^. It has been reported that autophagy activity is reduced in IPF ^9, 10, 11, 12, 13^, however, signalling pathways leading to this phenomenon remain to be elucidated. Here, we report that inactivation of Liver Kinase B1 (LKB1, encoded by the gene *Serine/Threonine Kinase 11, STK11*) in ATII cells inhibits autophagy and induces EMT. We identified downregulation of *CAB39L*, the allosteric activator of LKB1 in IPF alveolar septae and found that levels of *CAB39L* were significantly inversely correlated with *SNAI2* (Snail2) suggesting that reduction of *CAB39L* in IPF alveolar epithelium leads to LKB1 inactivation and promotes EMT. The profibrotic relevance of alveolar LKB1 inactivation was demonstrated in 3D co-cultures of ATII cells and lung fibroblasts in which paracrine signalling between LKB1-depleted ATII cells and fibroblasts was shown to augment myofibroblast differentiation.

## Results

### Global transcriptomic changes in LKBl-depleted alveolar type II (ATII) cells

It was reported previously that activation of AMP-activated protein kinase (AMPK), a downstream effector of LKB1, in myofibroblasts from IPF lungs reduces fibrogenic activity ^14^. To determine if, and how, ATII cells responded to alteration of LKB1 activity, we characterized the global transcriptomic changes in ATII cells upon RNA interference (RNAi)-mediated LKB1 depletion by performing RNA-seq. The human ATII cell line grows in continuous culture and expresses the ATII cell marker, pro-surfactant protein C, as reported earlier ^7^.

**Figure 1:**
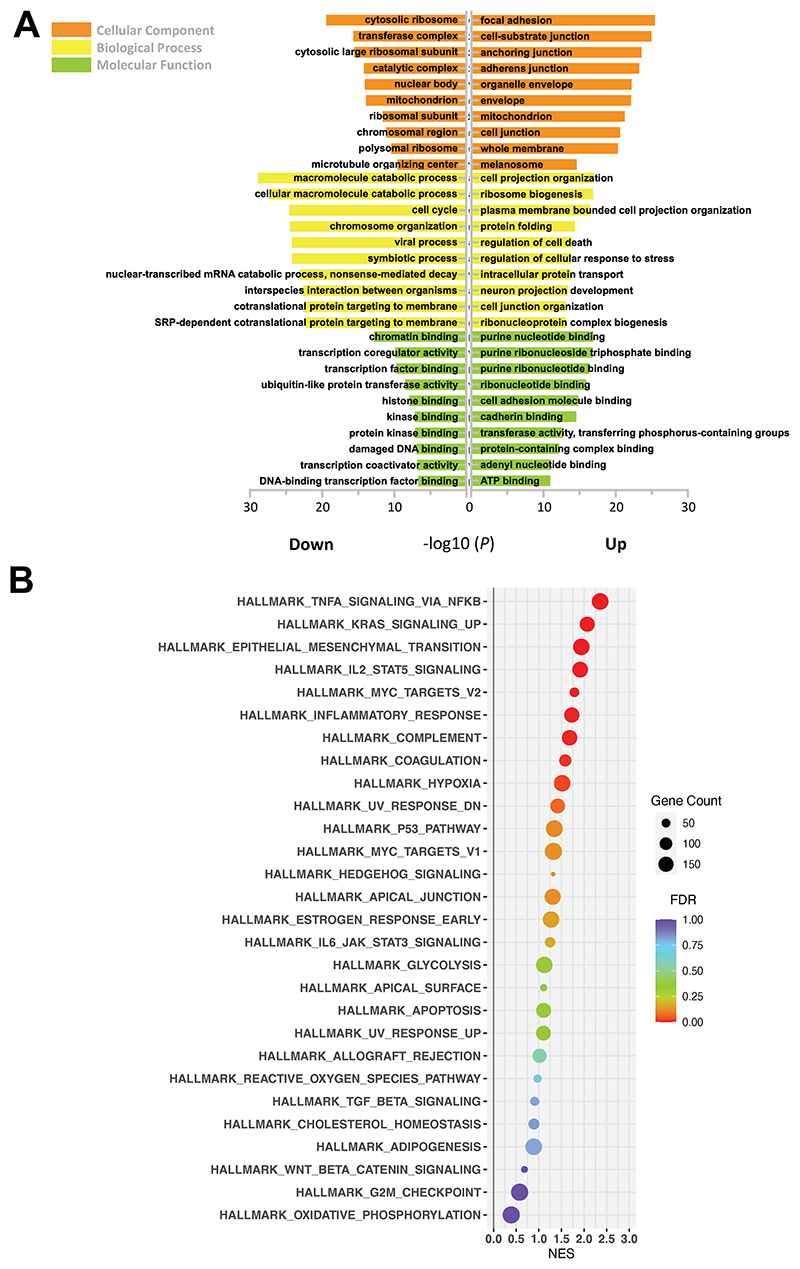
Global transcriptomic changes in LKB1-depleted ATII cells. **A.** Bar plot showing Gene Ontology (GO) enrichment of upregulated and downregulated differentially expressed genes (DEGs) in 3 groups: cellular component (orange), biological processes (yellow), and molecular functions (green). The top 10 enriched GO terms are arranged in -Log_10_(*P*-value). **B.** Scatter plot showing Gene Set Enrichment Analysis (GSEA). Results are ranked by the normalised enrichment score (NES). The colour and size of the dots represent false discovery rate (FDR) and gene counts, respectively.

Differentially expressed genes (DEGs) were defined by a false discovery rate (FDR)-adjusted *P* value (*P*adj) less than 0.05 and |Log2FoldChange| above 1. In total, 763 up-regulated and 664 down-regulated DEGs were identified (Table S1). Gene Ontology (GO) enrichment analysis was performed and grouped into molecular function, biological process and cellular component. Of note, several EMT-related terms were identified, including cell junction, chemotaxis and regulation of cell migration (FDR < 0.05; Figs. 1A and S1; Table S2). To provide further mechanistic insights, Gene Set Enrichment Analysis (GSEA) ^15^ was performed and several Hallmark pathways were identified, including "TNFα signalling pathway via NFkB" and "EMT" as top up-regulated pathways in LKB1-depleted ATII cells (Fig 1B; Table S3).

### LKBl depletion in ATII cells induces EMT

**Figure 2:**
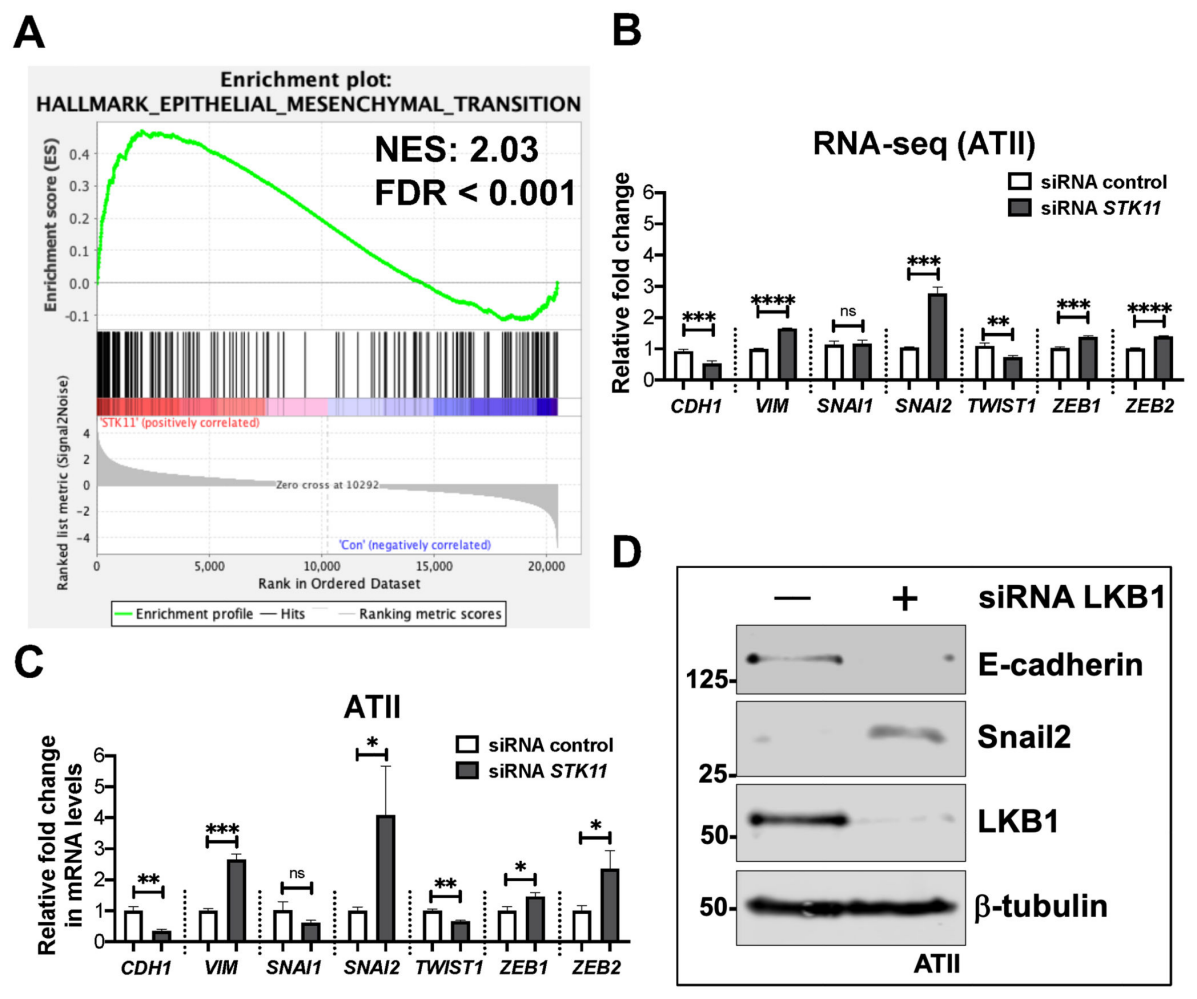
LKB1 depletion in ATII cells induces EMT. **A.**Gene Set Enrichment Analysis (GSEA) plot showing the enrichment of Hallmark_Epithelial-Mesenchymal Transition in LKB1-depleted ATII cells. Normalised enrichment score (NES) and false discovery rate (FDR) are indicated. **B.**RNA-seq data showing relative expressions of *CDH1* (encoding E-cadherin), *VIM* (encoding Vimentin), *SNAI1* (encoding Snail1), *SNAI2* (encoding Snail2), *TWIST1, ZEB1* and *ZEB2* in LKB1-depleted ATII cells *vs*. control. Data are mean ± s.d.; n = 3 samples in each group. **P < 0.01; ***P < 0.001; ****P < 0.0001 and ns: not significant. **C.**Relative fold changes in mRNA levels of *CDH1* (E-cadherin), *VIM* (Vimentin), *SNAI1* (Snail1), *SNAI2* (Snail2), *TWIST1, ZEB1* and *ZEB2* in LKB1-depleted ATII cells *vs*. control. *ACTB* (encoding β-actin)-normalised mRNA levels in ATII cells were used to set the baseline value at unity. Data are mean ± s.d.; n = 3 samples in each group. *P < 0.05; **P < 0.01; ***P < 0.001 and ns: not significant. **D.**Protein expressions of E-cadherin, Snail2, and LKB1 in ATII cells transfected with the indicated siRNA. β-tubulin was used as a loading control.

Given that the "Hallmark-EMT" pathway was positively enriched upon LKB1 *(STK11)* depletion in ATII cells (Fig. 2A; normalized enrichment score, NES = 2.03; FDR < 0.001), we examined changes in EMT-associated genes. We identified increases in expression of *VIM* (Vimentin, a mesenchymal marker) and several EMT-transcriptional factors, in particular *SNAI2* (encoding Snail2), as well as a reduction in *CDH1* (encoding E-cadherin, an epithelial marker) in our RNA-seq dataset (Fig. 2B) which we confirmed by real time qPCR (Fig. 2C), as well as demonstrating increased protein levels of Snail2 while E-cadherin protein was decreased (Fig. 2D). Together, these results demonstrate that loss of LKB1 activates an EMT programme in ATII cells.

### LKB1 depletion leads to autophagy inhibition-mediated EMT via the p62-NFκB-Snail2 pathway in ATII cells

**Figure 3:**
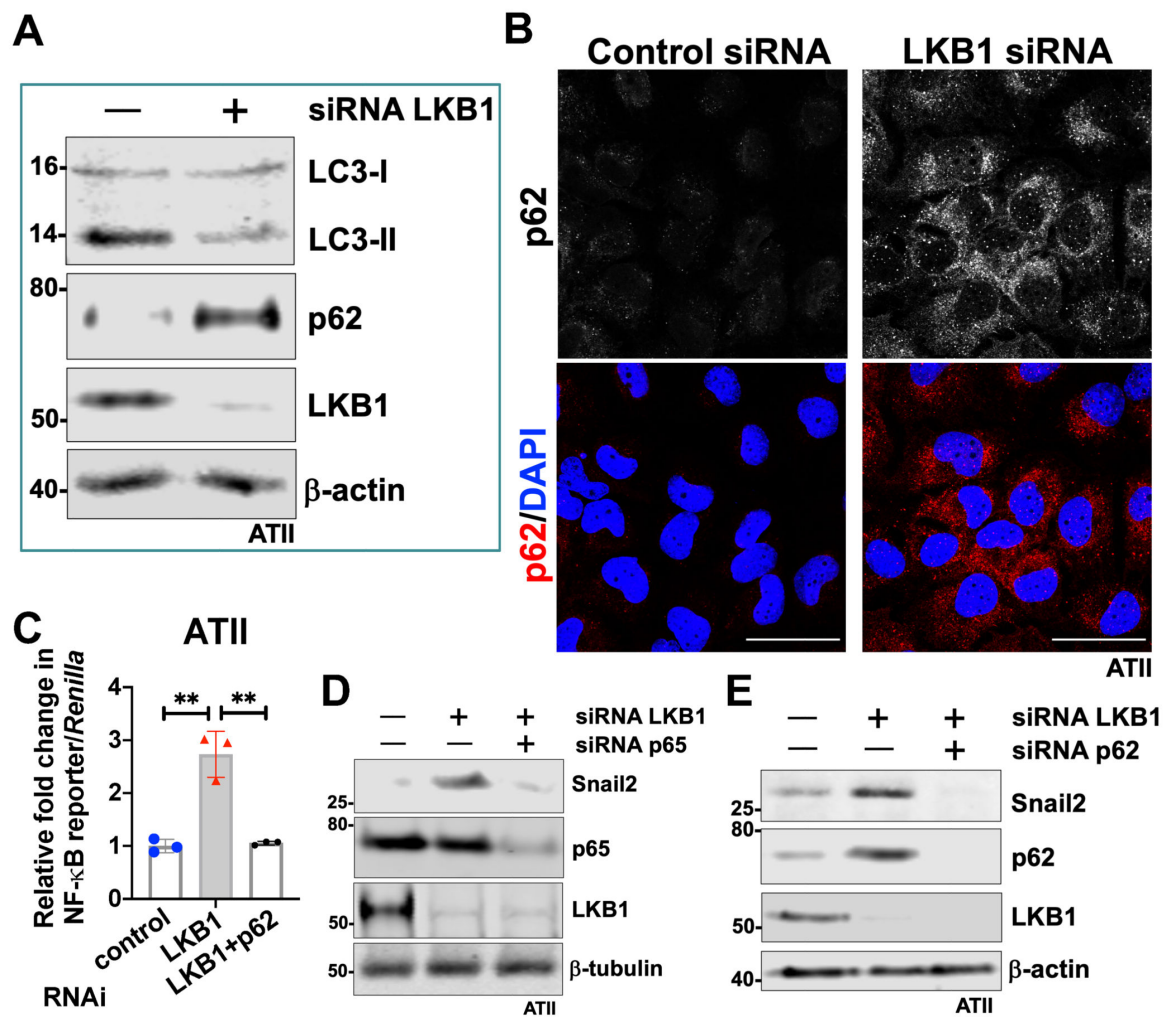
LKB1 depletion leads to autophagy inhibition-mediated EMT via the p62- NFκB-Snail2 pathway in ATII cells. **A.** Protein expressions of LC3-I, LC3-II, p62 and LKB1 in ATII cells transfected with the indicated siRNA. β-actin was used as a loading control. **B.** Immunofluorescence staining of p62 (red) in ATII cells transfected with the indicated siRNA. DAPI (blue) was used to stain nuclei. Scale bar: 40 μm. **C.** NF-kB reporter assays in ATII cells with the indicated treatment. Values represent the relative fold of *Renilla* luciferase, normalised against control (1.0). Data are mean ± s.d.; n = 3 samples in each group. **P<0.01. **D.** Protein expression of Snail2, p65 and LKB1 in ATII cells with the indicated treatment. β-tubulin was used as a loading control. **E.** Protein expression of Snail2, p62 and LKB1 in ATII cells with the indicated treatment. β-actin was used as a loading control.

Another highly enriched pathway in LKB1-depleted ATII cells was "Hallmark-TNF" signalling pathway via NFκB" (Fig. S2A; normalized enrichment score, NES = 2.45; FDR < 0.001). To verify this, we assessed NFκB activity using a reporter assay identifying that LKB1 depletion in ATII cells increased NFκB activity above 2-fold (Fig. S2B; *P* < 0.01). We have previously demonstrated that autophagy inhibition induced accumulation of p62/*SQSMT1* and activation of the NFκB pathway ^3, 16^ . Given our identification of increased NFkB activity upon LKB1 silencing, we therefore investigated the role of LKB1 on autophagy activity in ATII cells. This identified that LKB1 depletion in ATII cells led to autophagy inhibition, as demonstrated by decreased levels of LC3-II and increased p62 by western blot analysis (Fig. 3A), as well as punctate staining for p62 by immunofluorescence (Fig. 3B).

We next checked if LKB1 depletion induced EMT via the p62-NFκB-Snail2 pathway. Depletion of p62 abolished the increase in NFκB activity induced by LKB1 knockdown (Fig. 3C), suggesting that LKB1 depletion in ATII cells triggers the NFκB pathway via p62. Functionally, knockdown of either NFκB p65 (Fig. 3D) or p62 (Fig. 3E) abolished the increase in Snail2 expression induced by LKB1 depletion in ATII cells. Taken together, these results demonstrate that LKB1 inactivation in ATII cells inhibits autophagy and promotes EMT via a p62-NFκB pathway.

### Down-regulation of *CAB39L* in human IPF lungs

**Figure 4:**
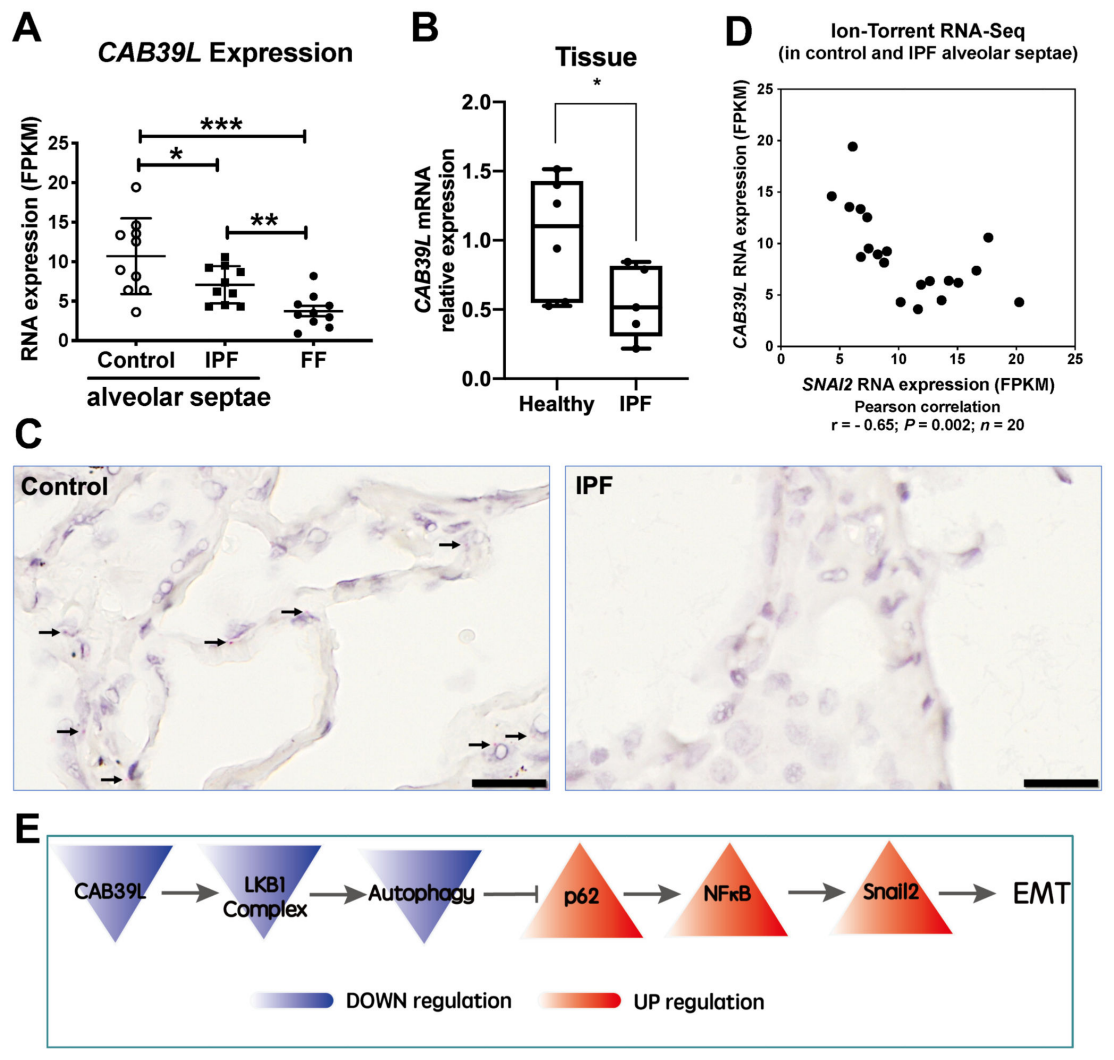
Down-regulation of *CAB39L* in human IPF lungs. **A.** Expression of *CAB39L* in healthy (control) alveolar septae, IPF alveolar septae and IPF fibroblast foci (n = 10 individual healthy and IPF donors; GSE169500). Relative expression levels are calculated as Fragments Per Kilobase of transcript per Million mapped reads (FPKM). Data are mean ± s.d.; n = 10 samples in each group. *P < 0.05; **P < 0.01 and ***P < 0.001. **B.** Relative fold changes in the mRNA level of *CAB39L* in human non-fibrotic control v*s*. IPF lung tissue. Data are mean ± s.d.; n = 6 samples in each group. *P < 0.05. **C.** Representative images of mRNA expression of *CAB39L* (arrows) in non-fibrotic control or IPF lung tissue using RNAscope® RNA *in-situ* hybridisation. Scale bar: 20 μm. **D.** Scatter plot to compare the expression of *CAB39L* and *SNAI2* (Snail2) in alveolar septae from non-fibrotic control and IPF lung tissue. (Pearson coefficient r = - 0.65; *P* = 0.002; n = 20). **E.** Diagram showing that *CAB39L* downregulation in IPF inactivates LKB1 complex, leading to autophagy inhibition-mediated EMT via the p62-NFκB-Snail2 pathway in ATII cells.

Activation of LKB1 occurs via allosteric binding of LKB1 to STE20-related adaptor *(STRAD)* and mouse protein 25 (MO25, encoded by *CAB39* and *CAB39L)* ^17^. Given our *in vitro* findings, we compared the expression of LKB1 *(STK11), STRADA, STRADB, CAB39* and *CAB39L* in IPF and control lungs in a transcriptomic dataset that we have recently established (GSE169500) ^18^. Briefly, laser capture microdissection was performed upon Formalin-Fixed Paraffin-Embedded (FFPE) control non-fibrotic lung tissue (alveolar septae, n = 10) and usual interstitial pneumonia/idiopathic pulmonary fibrosis FFPE lung tissue (fibroblast foci and adjacent non-affected alveolar septae, n = 10 each), followed by RNA-seq. Among those subunits within the LKB1 complex, only the expression of *CAB39L*, the allosteric activator of LKB1, was down-regulated in IPF alveolar septae (Fig. 4A). By contrast, expression of other subunits, including LKB1 *(STK11)*, *STRADA*, *STRADB* and *CAB39*, did not significantly change in IPF lungs (Fig. S3). Down-regulation of *CAB39L* in human IPF lung tissue was confirmed using real time qPCR of IPF tissue lysates (Fig. 4B; *P* < 0.05) as well as RNA *in situ* hybridisation of alveolar septae (Fig. 4C). We also assessed the expression of *CAB39L* and *SNAI2* (encoding Snail2) in alveolar septae from control and IPF lungs using the same dataset (GSE169500) and found that the levels of *CAB39L* were significantly inversely correlated with *SNAI2* (Snail2) (Fig. 4D, Pearson r = - 0.65; n = 20; *P* = 0.002). These observations support the concept that in IPF alveolar epithelium down-regulation of *CAB39L* leads to LKB1 inactivation and promotes EMT via the p62-NFκB pathway (Fig. 4E).

### 3D co-cultures of ATII cells and pulmonary fibroblasts suggest involvement of paracrine signalling in augmenting myofibroblast differentiation

**Figure 5:**
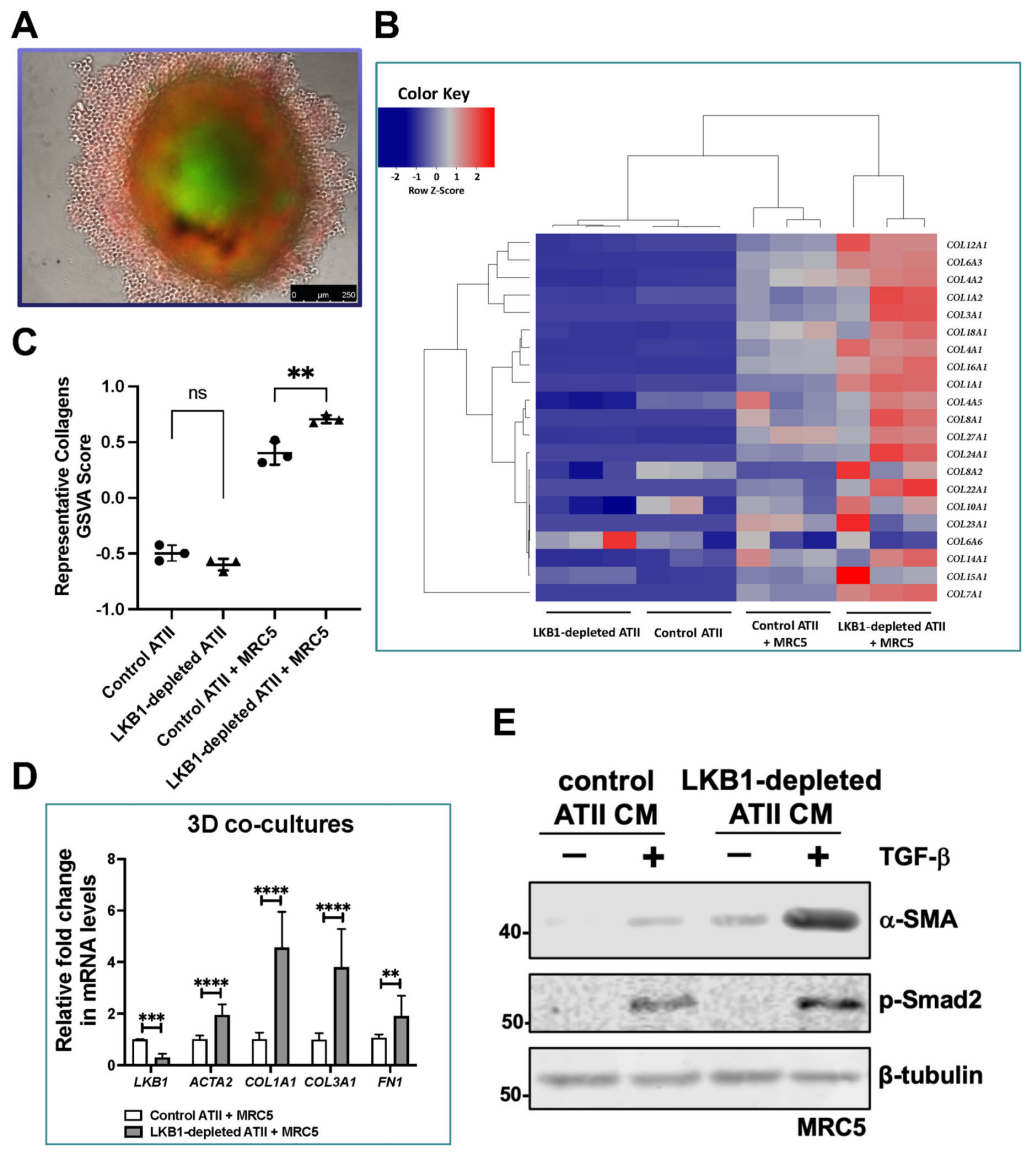
3D co-cultures of ATII cells and MRC5 coupled with RNA-seq suggest a role of paracrine signalling in augmenting myofibroblast differentiation. **A.** A representative image showing a 3D co-culture spheroid of MRC5 lung fibroblasts (green) and ATII cells (red). Scale bar: 250 μm. **B.** Heatmap and hierarchical cluster analysis of multiple collagen genes in 2D-cultured control or LKB1-depleted ATII cells and 3D co-cultures of MRC5 with control or LKB1-depleted ATII cells. Red indicates up-regulation and blue down-regulation. n = 3 samples in each group. **C.** Graph showing Gene Set Variation Analysis (GSVA) scores using a collagen signature in 2D-cultured control or LKB1-depleted ATII cells, and 3D co-cultures of MRC5 with control or LKB1-depleted ATII cells. Data are mean ± s.d.; n = 3 samples in each group. **P <0.01 and ns: not significant. **D.** Relative mRNA expressions of *STK11* (encoding LKB1)*, ACTA2* (encoding α-SMA), *COL1A1*, *COL3A1* and *FN1* in the spheroid samples from MRC5 co-cultured with control or LKB1-depleted ATII cells. *ACTB* (encoding β-actin)-normalised mRNA levels in ATII cells were used to set the baseline value at unity. Data are mean ± s.d.; n = 3 samples in each group. **P < 0.01; ***P < 0.001 and ****P < 0.0001. **E.** Protein expression of α-SMA and phospho-Smad2 (p-Smad2) in MRC5 with indicated treatments. β-tubulin was used as a loading control.

Our previous study reported that reduced autophagy activity contributed to fibrosis via aberrant epithelial-fibroblast crosstalk ^3^. To determine if it was also the case for LKB1-depletion in ATII cells, 2D cultures of ATII cells alone or 3D co-cultures with MRC5 lung fibroblasts (Fig. 5A) were established and then analysed by RNA-seq. DEGs were defined by a FDR-adjusted *P* value (*P*_adj_) less than 0.05 and |Log_2_FoldChange| above 1 (Table S4).

Our earlier reports ^3, 5^ by comparison of the relative expression of ECM components in ATII cells and fibroblasts highlight that ATII cells produce extremely low levels of ECM genes even after the induction of EMT, suggesting that ECM production in fibrosis is more likely to be a consequence of fibroblast activation than direct deposition by epithelial cells undergoing EMT. This is also true in this study. A comparison of collagen genes in 2D monocultures of control or LKB1-depleted ATII showed relatively low levels of collagen gene expression that were not significantly different (control ATII *vs*. LKB1-depleted ATII; Fig. 5B; Table S5), even though an EMT signature was clearly detectable in the LKB1 depleted cells (Figs. 1 and 2). In contrast, when LKB1-depleted ATII cells were co-cultured with MRC5 fibroblasts, there was a marked up-regulation of a large number of collagen genes (control ATII + MRC5 *vs*. LKB1-depleted ATII + MRC5; Fig. 5B; Table S5). Quantification of the changes in collagen gene expression using Gene Set Variation Analysis (GSVA) ^19^, identified a significant effect of LKB1 depletion in 3D co-cultures of ATII cells and MRC5 (Fig. 5C; *P* < 0.01), but not in 2D-cultured ATII cells (Fig. 5C; *P* > 0.05). This suggested collagen production in IPF lungs is unlikely to be a direct consequence of epithelial collagen gene expression due to EMT, but rather epithelial cells exhibiting an indirect effect on fibroblast differentiation via paracrine signalling, especially when undergoing EMT ^3, 5^.

To further explore the effect of paracrine signalling on fibroblast to myofibroblast transition, in 3D co-cultures we analysed the expression of *ACTA2* (α-SMA, a myofibroblast marker), confirming that LKB1 depletion in ATII cells caused increased expression and that this was associated with up-regulations in *COL1A1, COL3A1*, and *FN1* (Fig. 5D; all *P values* < 0.01). Finally, to confirm the paracrine influence of ATII cells on fibroblast differentiation, we treated MRC5 cells with conditioned media (CM) from ATII cells transfected with control or LKB1 siRNA without or with addition of transforming growth factor-β (TGF-β), and assessed levels of α-SMA. CM from ATII cells transfected with LKB1 siRNA without TGF-β had a similar effect on α-SMA expression compared to TGF-β treatment alone (Fig. 5E). Furthermore, CM from LKB1-depleted ATII cells together with TGF-β achieved a strong synergistic effect on α-SMA protein levels (Fig. 5E). These data suggest that LKB1 depletion in ATII cells augments myofibroblast differentiation via paracrine signalling.

## Discussion

IPF is a progressive interstitial lung disease with limited treatment options available ^20^. Although the underlying cause of IPF is not fully understood, repetitive microinjuries to aged alveolar epithelium is proposed to trigger aberrant wound healing processes, initiating an accumulation of ECM deposited by myofibroblasts ^21^, which are critical in the pathogenesis of IPF, with increased fibroblast foci associated with worse prognosis ^22^. The origin of myofibroblasts in IPF is controversial and it was proposed that ATII cells undergoing EMT may be a source of myofibroblasts in fibrotic diseases. However, findings from our group suggest that ATII cells undergoing EMT induced by RAS activation ^5^ or autophagy inhibition ^3^ express only low levels of ECM genes. Our findings in this study support the concept that epithelial cells do not directly contribute to myofibroblast populations via EMT, rather they are able to promote myofibroblast differentiation through paracrine signaling ^7^.

LKB1 is an evolutionarily conserved serine/threonine protein kinase, which acts as an important regulator of cell polarity, proliferation, and cell metabolism in epithelial cells ^23^. Activation of LKB1 activation occurs via allosteric binding of LKB1 to STE20-related adaptor *(STRAD’)* and mouse protein 25 (MO25, encoded by *CAB39* and *CAB39L*) ^24^ Many of the best-known functions of LKB1 are attributable to its ability to activate AMPK, which is an important conserved regulator of cell growth and metabolism ^25^. It was reported recently that activation of AMPK in myofibroblasts from IPF lungs displays lower fibrotic activity. In a bleomycin mouse model of lung fibrosis, metformin accelerates the resolution of well-established fibrosis in an AMPK-dependent manner ^14^. This study supports a role for such an approach to reverse established fibrosis by facilitating deactivation and apoptosis of myofibroblasts ^14^. In line with this, a recent study suggests that in patients with IPF and type 2 diabetes, metformin therapy may be associated with improved clinical outcomes. However, further investigation with randomized clinical trials is necessary prior to metformin's broad implementation in the clinical management of IPF ^26^.

Han and colleagues reported that kidney-specific deletion of *Lkb1* induces severe renal fibrosis ^27^. Similar to our findings, they found LKB1 *(STK11)* mRNA levels are not statistically significant altered in fibrotic kidney samples. Instead, the expression of the allosteric activator of LKB1, *CAB39L*, is significantly decreased in kidney fibrosis ^27^, raising its potential role in the development of fibrotic disease. Coincidently, thymoquinone alleviates thioacetamide-induced hepatic fibrosis by activating the LKB1-AMPK signaling pathway in mice ^28^. Apart from AMPK, LKB1 also activates a family of 12 "AMPK-related kinases", including BRSK1, BRSK2, NUAK1, NUAK2, QIK, QSK, SIK, MARK1, MARK2, MARK3, MARK4 and MELK ^29^. It was shown earlier that LKB1 suppresses EMT-transcriptional factor Snail1 ^30^ and ZEB1 ^31^ expression via MARK1/4 and miR-200a/c, respectively. In this study, we demonstrated that LKB1 depletion induces Snail2 expression via autophagy inhibition-p62-NFκB pathway in ATII cells, consistent with our previous reports ^3, 16^.

As a highly conserved process ^32^, autophagy has been associated with several human diseases, including pulmonary fibrosis (see our recent review ^33^). It has been reported that LKB1 modulates autophagy activity via an AMPK-mTORC1 ^34, 35^ or AMPK-ULK1 (ATG1) axis ^36^. In ATII cells, upon LKB1 inhibition, autophagy activity is reduced, leading to EMT via the p62-NFκB pathway (Fig. 4E). This drives local myofibroblast differentiation via paracrine signalling.

In summary, this study provides novel insights into the role of epithelial LKB1 in pulmonary fibrosis, highlighting the potential therapeutic intervention by targeting this pathway in IPF.

## Materials and Methods

### Cell Culture, reagents and transfections

Sources of cell lines, culture conditions and short interfering RNA (siRNA) transfections were reported earlier ^3, 4, 5, 16, 18, 37, 38^. MRC5 lung fibroblasts were obtained from the European Collection of Authenticated Cell Cultures (ECACC). Fibroblasts were cultured in Dulbecco’s Modified Eagle’s Medium (DMEM) supplemented with 10% foetal bovine serum (FBS), 50 units/ml penicillin, 50μg/ml streptomycin, 2mM L-glutamine, 1mM sodium pyruvate, and 1x non-essential amino acids (all from Life Technologies). An alveolar type II (ATII) cell line ^39, 40, 5^ was cultured in DCCM-1 (Biological Industries Ltd) supplemented with 10% new-born calf serum (NBCS) (Life Technologies), 1% penicillin, 1% streptomycin, and 1% L-glutamine (all from Life Technologies). The human ATII cell line grows in continuous culture and expresses the ATII cell marker, pro-surfactant protein C (ProSP-C) ^3, 5^. All cells were kept at 37 °C and 5 % CO_2_. All cultures were tested and free of mycoplasma contamination. Details are provided in the Supplementary Methods.

### Three-dimensional (3D) co-cultures

Aggregation of 3D co-cultures was achieved using Nanoshuttle-PL (Greiner Bio-One). Briefly, MRC5 fibroblasts at 80% confluence were treated with Nanoshuttle-PL for 24 h, before trypsinisation. Cells were then pipetted onto cell repellant 96-well plates sat on a magnetic drive and left to incubate at 37 °C and 5% CO_2_ for a minimum of 3 h on the magnetic drive to enable the spheroid to form. The process was then repeated for control or LKB1-depleted ATII cells so they could then grow around the existing fibroblast spheroid. LKB1 -depleted ATII cells were generated by transfection with LKB1 (*STK11*) siRNA oligos at a final concentration of 35 nM using DharmaFECT 2 reagent (Dharmacon).

### RNA *in situ* hybridization

*In situ* detection of *CAB39L* mRNA on formalin-fixed paraffin-embedded (FFPE) sections of lung tissue from patients with IPF or non-fibrotic control were performed using RNAscope® technology (Advanced Cell Diagnostics, Biotechne, Abingdon, UK) (n = 3 samples each group) ^18^. *CAB39L* mRNA was detected by the predesigned probe. Briefly, lung tissue sections (thicknesses: 5 μm) were baked at 60 °C, deparaffinized in xylene, followed by dehydration in graded ethanol. Target retrieval, hybridization with target probe, amplification, and chromogenic detection were performed according to the manufacturer’s recommendations (RNAscope 2.5 HD Assay-RED for FFPE tissues). Sections were counterstained with Gill’s Hematoxylin and mounted with Vectamount prior to imaging. Assays were performed with positive (Peptidylprolyl Isomerase B, *PPIB)* and negative controls. Images were acquired using an Olympus Scanner VS110 (Olympus UK, Southend-on-Sea, UK).

### Methods for RNA-seq and bioinformatics; western blot; real-time qPCR; immunofluorescence microscopy; luciferase reporter assay and statistical analysis

were reported earlier ^3, 4, 5 16, 18, 37, 38^, with details provided in the Supplementary Methods.

## Supplementary Material

Supple Table S1

Supple Table S2

Supple Table S3

Supple Table S4

Supple Table S5

Supple Text

## Data Availability

All data generated or analysed during this study are included in the manuscript and supporting files. The RNA-seq data have been deposited in the Gene Expression Omnibus (GEO) database(accession code GSE205970; the following secure token ifstmcyadrqvler has been created to allow review of this record while it remains in private status).
