# Supplementary material for: LKB1 depletion-mediated epithelial–mesenchymal transition induces fibroblast activation in lung fibrosis
Source: Genes Dis. Author manuscript; Available in PMC 2024 May 1. (PMC7615521; doi:10.1016/j.gendis.2023.06.034)
Supplement: Supple Text [file EMS177469-supplement-Supple_Text.pdf]

LKB1 inhibition in alveolar epithelial cells induces fibroblast activation in pulmonary fibrosis

Supplementary Materials

Table of contents

Supplementary Methods ***2***

1. Cell culture, reagents and transfections 2
2. Lung tissue sampling 2
3. RNA-seq and bioinformatic analysis 3
4. Western blot analysis 4
5. Real-time qPCR analysis 4
6. Immunofluorescence microscopy 4
7. Luciferase reporter assay 5
8. Statistical analysis 6

Supplementary Figures ***7***

Supplementary Figure 1. 7
Supplementary Figure 2 8
Supplementary Figure 3. 9
Supplementary Figure 4 10

Supplementary Tables ***11***

R Scripts ***12***

Raw data for western blots ***19***

Supplementary Methods

1. Cell culture, reagents and transfections

MRC5 lung fibroblasts were obtained from the European Collection of Authenticated Cell Cultures (ECACC). Fibroblasts were cultured in Dulbecco's Modified Eagle's Medium (DMEM) supplemented with 10% foetal bovine serum (FBS), 50 units/ml penicillin, 50µg/ml streptomycin, 2mM L-glutamine, 1mM sodium pyruvate, and 1x non-essential amino acids (all from Life Technologies). Alveolar type II (ATII) cells [1] [2] [3] were cultured in DCCM-1 (Biological Industries Ltd) supplemented with 10% new-born calf serum (NBCS) (Life Technologies), 1% penicillin, 1% streptomycin, and 1% L-glutamine (all from Life Technologies). All cells were kept at 37 °C and 5 % CO_2. All cultures were tested and free of mycoplasma contamination.

Short interfering RNA (siRNA) against *STK11* (LKB1) (M-005035-02-0010), *SQSTM1* (p62) (MU-010230-00-0002) and *RELA* (p65) (MU-003533-02-0002) were purchased from Dharmacon. Sequence is available from Dharmacon, or on request. ATII cells were transfected with the indicated siRNA at a final concentration of 35 nM using DharmaFECT 2 reagent (Dharmacon). siGENOME RISC-Free siRNA (Dharmacon) was used as a negative control.

2. Lung tissue sampling

All human lung tissue samples for primary cell culture were approved by the Southampton and South West Hampshire and the Mid and South Buckinghamshire Local Research Ethics Committees, and all subjects gave written informed consent. Clinically indicated IPF lung biopsy tissue samples and non-fibrotic control tissue samples (macroscopically normal lung sampled remote from a cancer site) were assessed as surplus to clinical diagnostic requirements. All IPF samples were from patients subsequently receiving a multidisciplinary diagnosis of IPF according to international consensus guidelines [4]

3. RNA-seq and bioinformatic analysis

RNA isolation and mRNA sequencing of samples were performed following the manufacturer's instructions (Novogene, UK). The ATII cells were treated transfected with siRNA against indicated siRNAs for 48h. Total RNA was isolated using RNeasy mini kit (Qiagen) according to manufacturer's instructions and quantified using a Nanodrop Spectophotometer 2000c (Thermo Fisher Scientific).

A total amount of 3 μg RNA per sample was used as input material for library construction. Sequencing libraries were generated using NEBNext® UltraTM RNA Library Prep Kit for Illumina® (NEB, Ipswich, Massachusetts, USA) following manufacturer's instruction. Libraries were pooled in equimolar and sequenced using the paired-end strategy (2 × 150) on the Illumina NovaSeq 6000 platform following the standard protocols (Novogene, UK). Raw read counts were imported into RStudio (version 4.2.0) and analyzed by using DESeq2 [5] (version 1.26.0). Transcripts with low abundance (under 10 counts across all samples) were removed. The R codes were provided in the Supplementary Materials. Genes with a false discovery rate (FDR) adjusted *P* value using Benjamini-Hochberg (BH) method less than 0.05 and $|Log_2FoldChange|$ above 1 were considered as differentially expressed genes (DEGs). DEGs in 2D-cultured LKB1-depleted ATII cells are provided in Table S1 and DEGs in 3D co-cultures of LKB1-depleted ATII cells and MRC5 in Table S4.

The collection of Hallmark gene sets generated from the gene set enrichment analysis (GSEA) software (version 4.1.0) (with registration) [6]. Gene Ontology (GO) terms and Hallmark enrichment analysis of DEGs were generated through DAVID website tools (https://david.ncifcrf.gov) with default parameters. FDR adjusted *P* values by Benjamini-Hochberg (BH) method were used to estimate the statistical significance. Details for GO enrichment analysis are provided in Table S2 and Gene Set Enrichment Analysis (GSEA) in Table S3.

A collagen gene score for each sample was calculated using Gene Set Variation Analysis (GSVA) based on a list of 21 collagen genes upregulated in 3D co-cultured LKB1-depleted ATII cells and MRC5 (Table S5).

4. Western blot analysis

Western blot analysis was performed with lysates from cells with Urea buffer (8 M Urea, 1 M Thiourea, 0.5% CHAPS, 50 mM DTT, and 24 mM Spermine). Primary antibodies were from: Santa Cruz (β-actin, sc-47778; Snail2, sc-10436), Abcam (β-tubulin, ab6046) and Cell Signalling Technology (α-SMA, 14968; Snail2, 9585; β-tubulin, 86298; LC3, 2775; p62/*SQSTM1*, 5114; p65/RELA, 8242; Phospho-Smad2, 3104). Signals were detected using Odyssey imaging system (LI-COR), and evaluated by ImageJ 1.42q software (National Institutes of Health).

5. Real-time qPCR analysis

Total RNA was extracted using RNeasy mini kit (Qiagen) following manufacturer's instructions. RNA concentration was quantified on a Nanodrop Spectophotometer 2000c (ThermoFisher Scientific, UK). Real-time PCR was carried out using gene-specific primers *SNAI1* (Snail1) (QT00010010), *SNAI2* (Snail2) (QT00044128), *ZEB1* (QT00008555), *ZEB2* (QT00008554), *TWIST1* (QT00011956), *STK11* (LKB1) (QT01008980), *ACTA2* (Alpha smooth muscle actin, α-SMA) (QT00088102), *COL1A1* (QT00037793), *COL3A1* (QT00058233) and *FN1* (QT00038024) or *ACTB* (β-actin) (QT01680476), QuantiNova SYBR Green RT-PCR kits (Qiagen) and QuantiTect Primer Assays(Qiagen) according to manufacturer's instruction or RNA was reverse transcribed and run with primers and Taqman probe sets obtained from Thermofisher Scientific, Reading UK. Relative transcript levels of target genes were normalised to *ACTB* (β-actin).

6. Immunofluorescence microscopy

The immunofluorescence assay was performed as previously described [7]. When the cells reached 80 - 90% confluency, media were removed and cells were gently washed with 1× PBS twice. One ml 4% paraformaldehyde (PFA) (Thermo Fisher Scientific, UK) in 1× PBS was added to fix the cells for 15 minutes. PFA was then removed and cells were washed with 1× PBS. For permeabilisation of cells, 500 µl of 0.1% TironX-100 (Thermo Fisher Scientific, UK) in 1× PBS was added to the each well of 12 well

plate and the slide was transferred from 6 well plate into 12 well plate and incubated in 0.1% TironX-100 for 5 minutes on ice. This was followed by washing the slides with 1× PBS twice. Then, cells on the slides were blocked in 0.2% Fish Skin Gelatine (Sigma Aldrich, UK) in 1× PBS for 60 minutes at room temperature. Meanwhile, anti-p62 primary antibody was prepared in blocking buffer with 1:50 dilution and paraflim was put on the foil wrapped container. To moisture the container, wet tissues were put into the side of box. Primary antibody was put on the paraflim and the excess buffer was got rid of from the slides and slides were put on the antibody upside down for 60 minutes at room temperature. 60 minutes after the primary antibody incubation (anti-p62/SQSTM1; Progen Biotechnik GmbH, GP62-C; 1:300), slides were flipped and put into 12 well plate and washed with 1× PBS 3 times, each time for 15 minutes on the rocker. Then, secondary antibody with 4'6-Diamidino-2-Pheylindole (DAPI) (Invitrogen, UK) was prepared in 1× PBS with the dilution of 1:400 and 1:1000, respectively. New paraflim was put into the box, 95 µl of secondary antibody was put onto paraflim, and slides were put onto paraflim upside down and incubated at room temperature for 60 minutes. Slides were washed with 1× PBS as previously by avoiding light. 8 µl of mounting solution was added to the cover slip and slide was put on the cover slip upside down and left to air dry overnight by avoiding light. Protein expression was detected using Alexa Fluor (1:400, Molecular Probes) for 20 minutes. Immunostained cells were analyzed and photographed using an Olympus IX83 inverted fluorescence microscope.

7. Luciferase reporter assay

The luciferase reporter assay was performed as previously described [3]. Cells were transfected using Lipofectamine 3000 (Life technology) in a 96-well plate with 100 ng of *Renilla* along with 100 ng of NFkB reporter per well. Cells were washed with 1 × PBS and lysed by trypsin (0.05% trypsin, Gibco), then centrifuged at 500 g for 5 minutes. Cell pellet was then re-suspended in certain amount of complete media before plating on a 96-well plate (Usually 100 µl of medium for each well) at 70-80% confluency. For each well to be transfected, 0.1 µl of Lipofectamine 3000 reagent (Life

technology) was diluted in 5 µl of Opti-MEM medium. Mixed well reagent was made and short vortexed. Diluted plasmids and Lipofectamine 3000 were mixed by pipetting up and down and the lipid-DNA mixture was incubated at room temperature for 15 minutes. Cells were transfected at 37°C for 48 hours before analysis. The transcriptional assay was carried out using the Dual-Luciferase reporter assay system (Promega, UK) following the manufacturer's protocol. Cells were wash with 1 × PBS prior to lysis in a 96-well plate. Cells were lysed in 100 µl of passive lysis buffer and put on a room temperature shaker for 15 minutes. Freezing lysates at -20 °C facilitated the lysis. Five µl of lysate was analyzed for each well in a 96 well white plate. Triplets were used for each transfection and 25 µl LAR II was first added and mixed by pipetting to measure the firefly luciferase activity. Another 25 µl of stop and go reagent was then added to help identify the *Renilla* activity. The final Dual-Luciferase Reporter activity was normalized based on both measurements.

8. Statistical analysis

Statistical analyses were performed in GraphPad Prism v7.02 (GraphPad Software Inc, San Diego, CA) unless otherwise indicated. No data were excluded from the studies and for all experiments, all attempts at replication were successful. For each experiment, sample size reflects the number of independent biological replicates and is provided in the figure legend. Normality of distribution was assessed using the D'Agostino-Pearson normality test. Statistical analyses of single comparisons of two groups utilised Student's *t*-test or Mann-Whitney *U*-test for parametric and non-parametric data respectively. Where appropriate, individual t-test results were corrected for multiple comparisons using the Holm-Sidak method. For multiple comparisons, one-way or two-way analysis of variance (ANOVA) with Dunnett's multiple comparison test or Kruskal-Wallis analysis with Dunn's multiple comparison test were used for parametric and non-parametric data, respectively. Results were considered significant if $P < 0.05$, where $*P < 0.05$, $**P < 0.01$, $***P < 0.001$, $****P < 0.0001$.

Supplementary Figures

Supplementary Figure 1. Global transcriptomic changes in LKB1-depleted ATII cells.

A. REVIGO TreeMap showing Gene Ontology (GO) analysis of upregulated differentially expressed genes (DEGs) in LKB1-depleted ATII cells. Common colours represent groupings based on parent GO terms, and each rectangle is a percentage of the relative enrichment of the GO term compared with the whole genome. Genes with a false discovery rate (FDR) less than 0.05 and |Log_2FoldChange| above 1 were considered as DEGs.

B. Scatter plot showing top ten GO terms enriched by upregulated DEGs in 3 functional groups: cellular component, biological processes and molecular functions. Rich factor is the percentage of DEG-enriched gene count in the given annotated GO terms. The sizes of circles represent gene counts, and the colours of circles represent FDR.

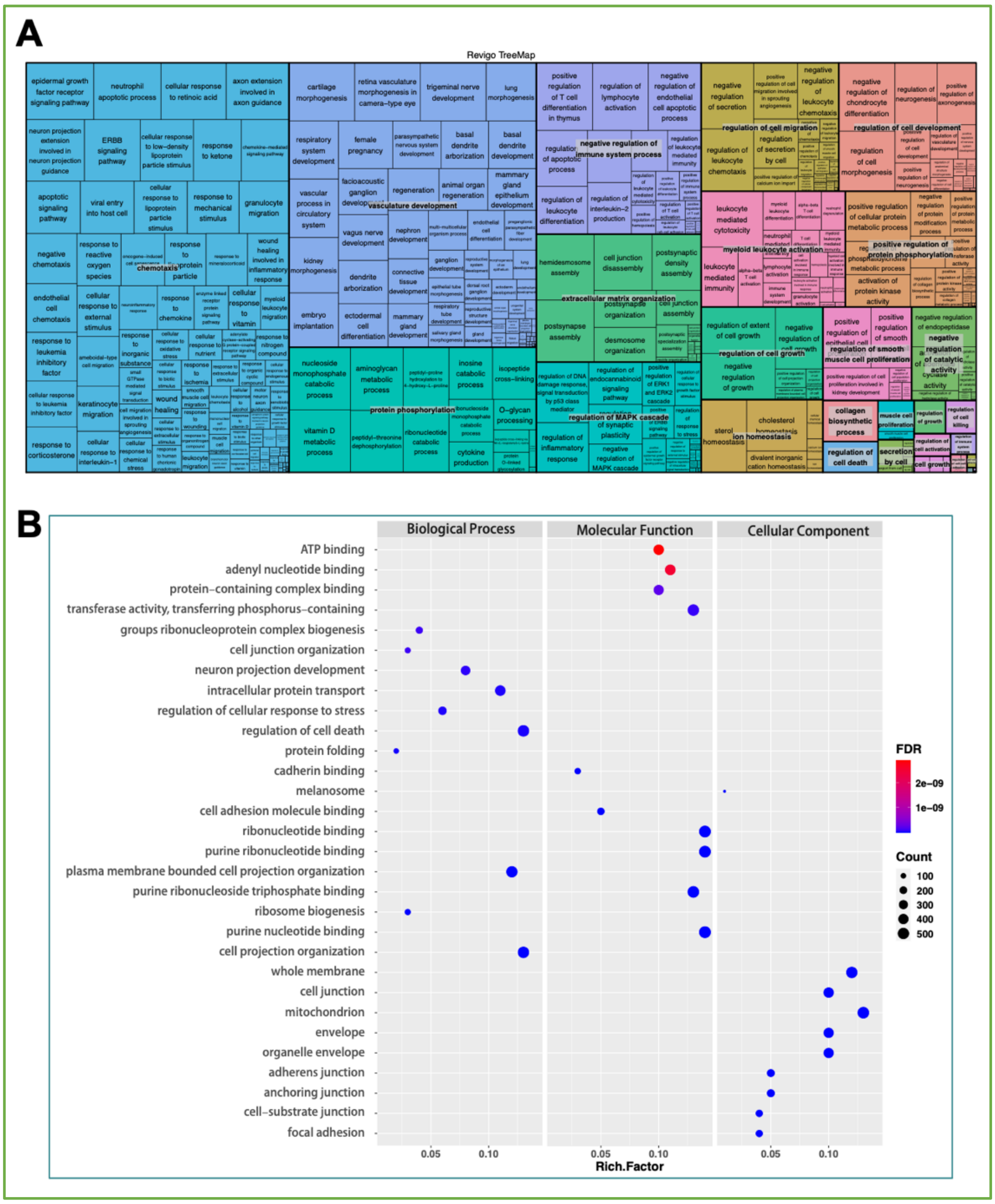

Supplementary Figure 2. LKB1 depletion leads to autophagy inhibition-mediated EMT via the p62-NFκB-Snail2 pathway in ATII cells.

A. Gene set enrichment analysis (GSEA) plot showing an enrichment of Hallmark_TNFA_Signaling_Via_NFKB in LKB1-depleted ATII cells. Normalised enrichment score (NES) and false discovery rate (FDR) are indicated.

B. NF-κB reporter assays in ATII cells transfected with the indicated siRNA. Values represent the relative fold of firefly luciferase in relation to Renilla luciferase, normalised against control (1.0). Data are mean ± s.d.; n = 3 samples in each group. $**P < 0.01$.

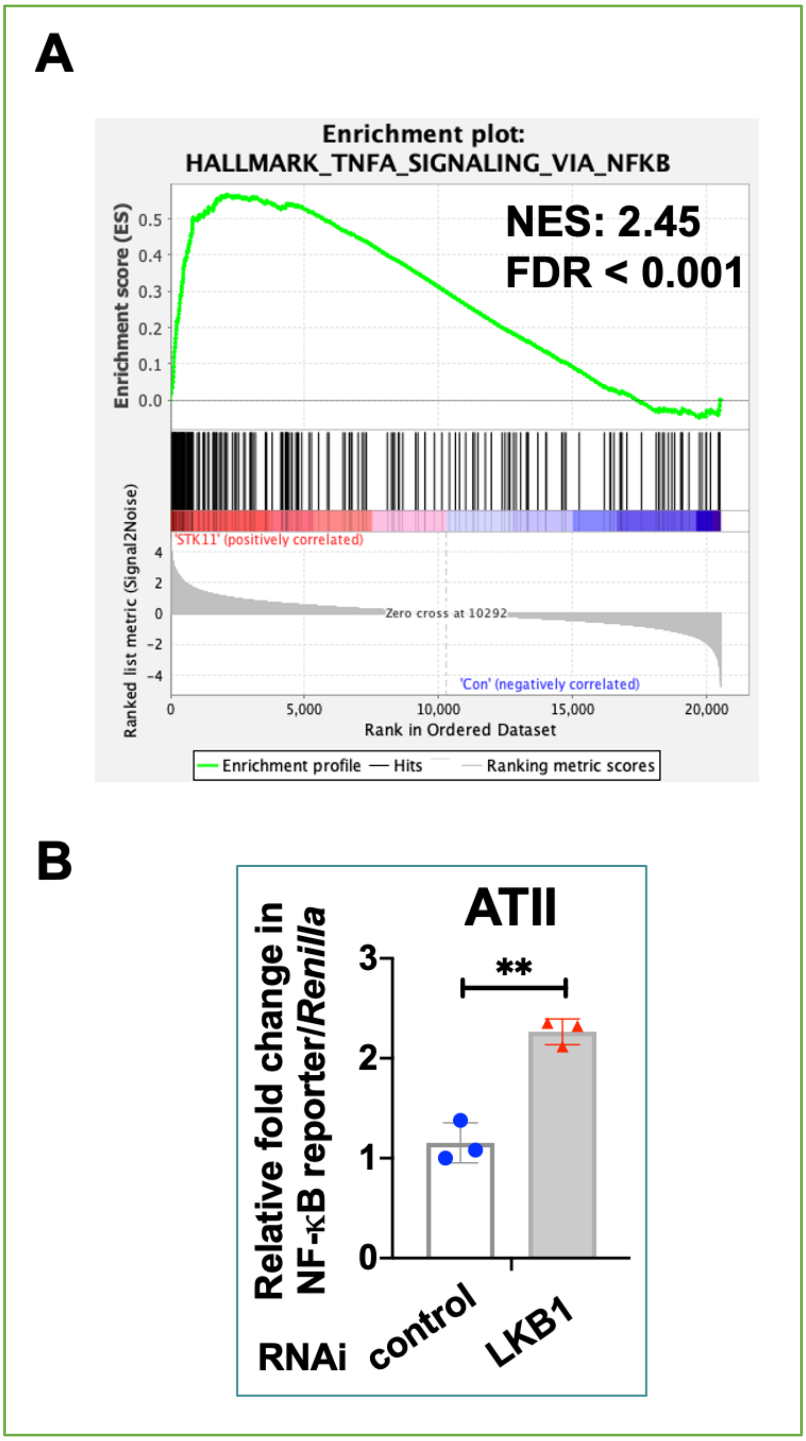

Supplementary Figure 3. **Down-regulation of *CAB39L* in human IPF lungs.** Expression of *STK11* (LKB1) (**A**), *STRADA* (**B**), *STRADB* (**C**) and *CAB39* (**D**) in healthy (control) alveolar septae, IPF alveolar septae and IPF fibroblast foci (n = 10 individual healthy and IPF donors; GSE169500). Relative expression levels are calculated as Fragments Per Kilobase of transcript per Million mapped reads (FPKM). Data are mean ± s.d.; n = 10 samples in each group.

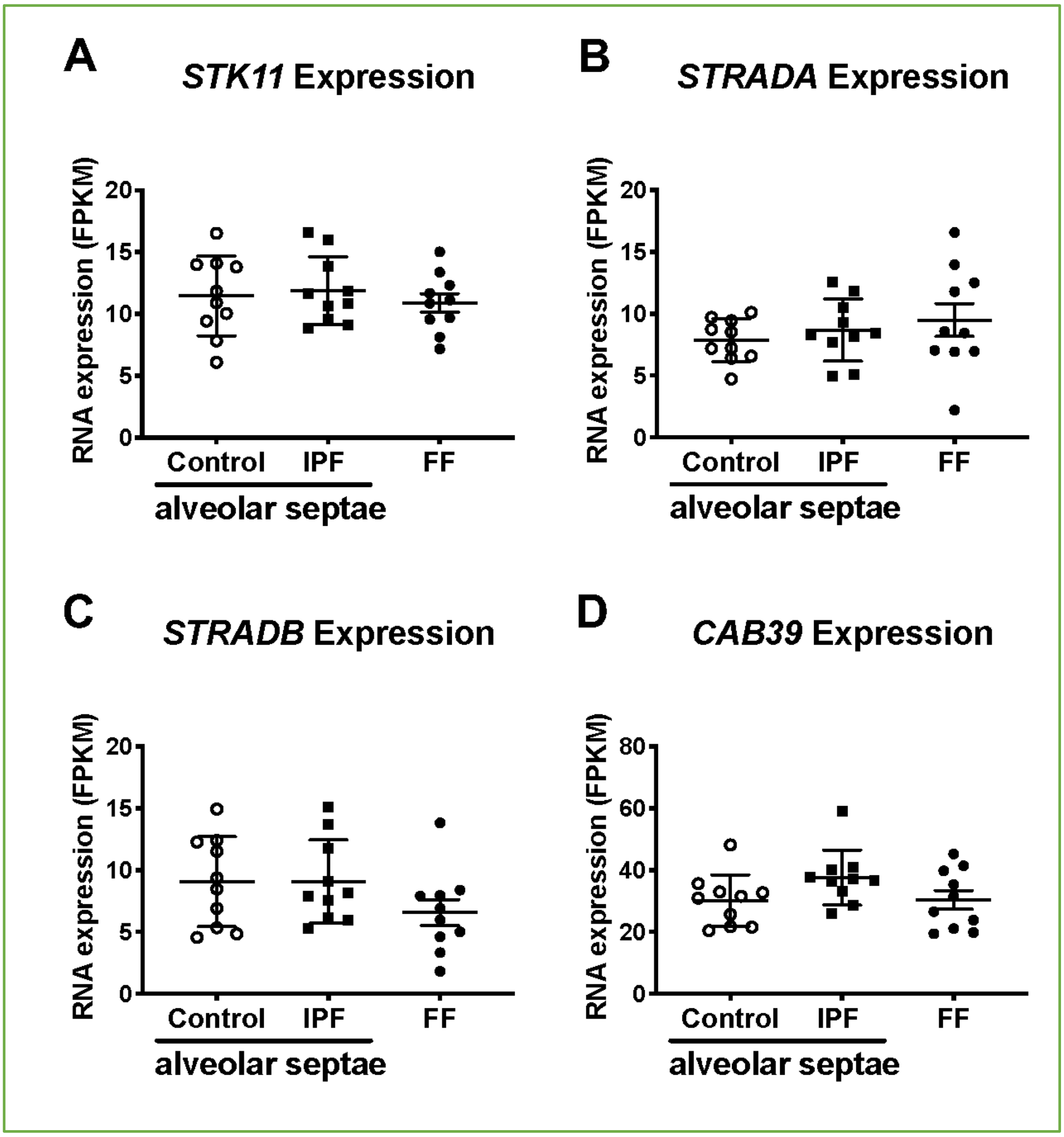

Supplementary Figure 4. 3D co-cultures of ATII cells and MRC5 coupled with RNA-seq suggest a role of paracrine signalling in augmenting myofibroblast differentiation.

A. Cell deconvolution based on an epithelial or a mesenchymal cell signature matrix derived from the single-cell RNA-seq (GSE135893) was used to determine cell compositions in spheroid samples from MRC5 co-cultured with control or LKB1-depleted ATII cells. Data are mean ± s.d.; n = 3 samples in each group. *$P < 0.05$.

B. RNA-seq data showing relative expressions of *ACTA2*, *COL1A1*, *COL3A1* and *FN1* in spheroid samples from MRC5 co-cultured with control or LKB1-depleted ATII cells. Data are mean ± s.d.; n = 3 samples per group. ****$P < 0.0001$ and ns: not significant.

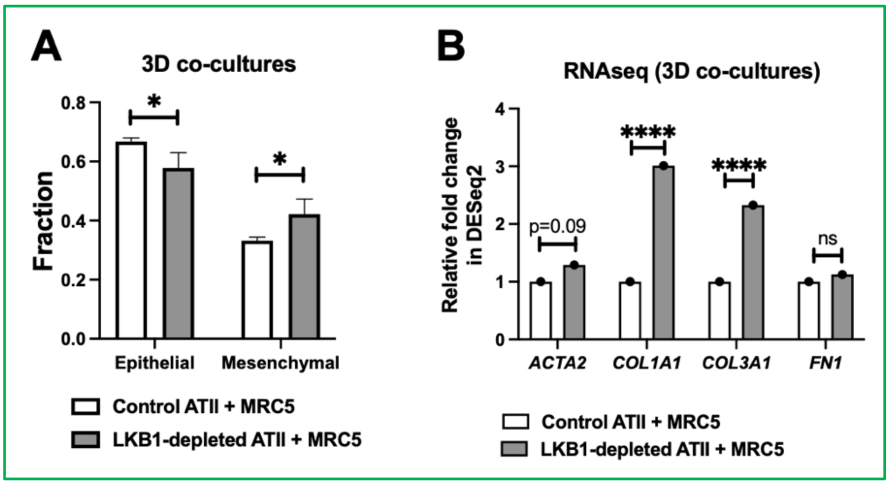

Supplementary Tables

Table S1. DEGs in LKB1-depleted alveolar type II (ATII) cells.

Table S2. GO terms enrichment analysis in LKB1-depleted ATII cells.

Table S3. GSEA in LKB1-depleted ATII cells.

Table S4. DEGs in 3D co-cultured LKB1-depleted ATII cells and MRC5.

Table S5. List of collagen genes used for GSVA calculation in Figure 5C.

R Scripts

Raw data were imported into RStudio (version 4.2.0). RStudio Version 1.4.1717 for IOS and R scripts were run.

```
# Set the working directory before run
# setwd("C:/Users/zx2n18/RNA-seq")
```

R codes for Figure 1B

```
library(ggplot2)
data <- read.csv ("Hallmark_gsea_Up.csv")
p <- ggplot(data, aes(NES, NAME))
p + geom_point(aes(colour=FDR.q.val, size=SIZE)) +
   scale_color_gradientn(colours=rainbow(4), limits=c(0, 1)) +
   geom_vline(xintercept=0, size=0.5, colour="gray50") +
   theme(axis.text = element_text(size = 8, face = "bold"),
panel.background=element_rect(fill="gray95", colour="gray95"),
            panel.grid.major=element_line(size=0.45,linetype='solid',
colour="gray90"),
            panel.grid.minor=element_line(size=0.45,linetype='solid',
colour="gray90"),
            axis.title.y=element_blank()) +
   expand_limits(x=c(0,3)) +
   scale_x_continuous(breaks=c(0,0.5,1,1.5,2,2.5,3)) +
   scale_y_discrete(limits=rev(data$NAME))
```

R codes for Figure 5B, S4A

```
#signature matrix building (scRNAseq by GSE13589)
library(dplyr)
source("https://z.umn.edu/archived-seurat")
library(Seurat)
library(patchwork)
install.packages('umap')
library(umap)
library(reticulate)
getwd()
setwd('/Users/lianyuanyuan/Desktop/Cell death and diease')
# Load the PBMC dataset
# Load the dataset
cell_type<-read.csv('/Users/lianyuanyuan/Library/Mobile
Documents/com~apple~CloudDocs/R/GSE135893_PF_subtypes/Control_Celltype.cs
v',header = T,sep = ',')
ppulmonary_fibrosis <- Read10X(data.dir = "/Users/lianyuanyuan/Library/Mobile
Documents/com~apple~CloudDocs/R/GSE135893_PF_subtypes/GSE135893_PF_su
btypes",gene.column = 1) ppulmonary_fibrosis_seurat <-
CreateSeuratObject(ppulmonary_fibrosis, project = "scRNA lung",min.features =
200) sc_data<-GetAssayData(object = pbmc.data, slot = 'counts')
sc_epi_mesenchymal<-sc_data[,cell_type$X]
#notice to delete the row.name with "march"or"sep" in the csv file, because that will
cause the "duplicate row.name" when you
#run it in the cibersorts.
write.table(sc_epi_mesenchymal,file='sc_epi_mesenchymal_Control_M.txt',sep =
'\t',row.names = T)
write.table(cell_type$population,file='sc_epi_mesenchymal_control_M_population.tx
t',sep = '\t',row.names = T)
```

R codes for Figure 5C

```
BiocManager::install("GSVA")
library("GSVA")
data <-read.csv("2d_3d_exprMatrix.rpm2.csv")
rownames(data)<-data[,1]
data<-data[,-1]
data<-as.matrix(data)
geneset<-read.csv("col_up_3d.csv")
GSVA <- gsva(data, geneSets, mx.diff=1)
```

R codes for Figure S1A

```
library(treemap)
revigo.names <-
c("term_ID","description","frequency","value","uniqueness","dispensability","represe
ntative");
revigo.data <- read.csv("GO_data.csv")
stuff <- data.frame(revigo.data);
names(stuff) <- revigo.names;
stuff$value <- as.numeric( as.character(stuff$value) );
stuff$frequency <- as.numeric( as.character(stuff$frequency) );
stuff$uniqueness <- as.numeric( as.character(stuff$uniqueness) );
stuff$dispensability <- as.numeric( as.character(stuff$dispensability) );
# by default, outputs to a PDF file
pdf( file="revigo_treemap.pdf", width=16, height=9 ) # width and height are in inches
# check the tmPlot command documentation for all possible parameters - there are a
lot more
treemap(
   stuff,
   index = c("representative","description"),
   vSize = "value",
   type = "categorical",
   vColor = "representative",
   title = "Revigo TreeMap",
   inflate.labels = FALSE,          # set this to TRUE for space-filling group labels -
good for posters
   lowerbound.cex.labels = 0,      # try to draw as many labels as possible (still, some
small squares may not get a label)
   bg.labels = "#CCCCCCAA",      # define background color of group labels
                                   # "#CCCCCC00" is fully transparent,
"#CCCCCCAA" is semi-transparent grey, NA is opaque
   position.legend = "none"
)
dev.off()
```

R codes for Figure S1B

```
mytheme <- theme(axis.title=element_text(face="bold", size=10,colour = 'gray25'),
axis.text=element_text(face="bold", size=10,colour = 'gray25'), axis.line =
element_line(size=0.5, colour = 'black'), panel.background =
element_rect(color='black'), legend.key = element_blank()
)
Go_Up<read.xlsx('/Toppgene_2dup_result.xlsx',sheet = 1)
Go_Up$Count<-sapply(Go_Up$InTerm_InList,function(x)
strsplit(x,"/")[[1]][1]) %>%as.numeric()
Go_Up$Member<-sapply(Go_Up$InTerm_InList,function(x)
strsplit(x,"/")[[1]][2])%>%as.numeric()
Go_Up$GeneRatio<-as.numeric(Go_Up[,10])/as.numeric(Go_Up[,11] )
Go_Up<-Go_Up[grepl('Member',Go_Up$GroupID),]
Go_Up$`P.adjust`<-10^(Go_Up$`Log(q-value)`)
Go_Up<-Go_Up[order(Go_Up$GeneRatio),]
Go_Up$Description<-factor(Go_Up$Description,levels = (Go_Up$Description))
#Plot
p<-ggplot(Go_Up,aes(GeneRatio,Description)) +

geom_point(aes(size=Count,color=`P.adjust`))+scale_colour_gradient(high='blue',low
='red',n.breaks=10)+
  theme_bw()+
  theme(axis.title=element_text(face="bold", size=10,colour = 'black'),
        axis.text=element_text(face="bold", size=10,colour = 'black'))
p
ggsave('Up_Go_result.pdf',width = 9,height = 8)
```

R codes for Figure S4B

```
library(gplots)
a1<-read.csv("colleagen_up.csv")
a1<-as.matrix(a1)
a1<-as.numeric(a1)
distCor <- function(a1) as.dist(1-cor(t(a1)))
hclustAvg <- function(a1) hclust(a1, method="average")
pdf("collagen_heatmap.pdf",width=20, height=20)# units="in", width=12, height=8,
res=300)
colorbar<-colorRampPalette(c('darkblue','grey','red'))(n=1000)
###set the color for heatmap
heatmap.2(a1, trace="none", density='none',margin=c(5,10),scale="row",cexRow =
1.3,
        #labRow = labels,
        cexCol = 0.8, zlim=c(-10,10),Colv = T,Rowv =
T,srtCol=45,adjCol=c(1,0),
        hclustfun = hclustAvg,distfun=distCor,symbreak=FALSE,key =
T,keysize = 1.5,
        #labRow =c(as.character(DEP_ras_expression_fc_pvalue$P.Value..)),

        #ColSideColors = condition_colors
        #RowSideColors=row_annotation)
)
dev.off()
```

References

1. Coelho, M.A., et al., *Oncogenic RAS signaling promotes tumor immunoresistance by stabilizing PD-L1 mRNA.* Immunity, 2017. **47**(6): p. 1083-1099. e6.
2. Molina-Arcas, M., et al., *Coordinate direct input of both KRAS and IGF1 receptor to activation of PI3 kinase in KRAS-mutant lung cancer.* Cancer discovery, 2013. **3**(5): p. 548-563.
3. Yao, L., et al., *Paracrine signalling during ZEB1-mediated epithelial–mesenchymal transition augments local myofibroblast differentiation in lung fibrosis.* Cell Death & Differentiation, 2019. **26**(5): p. 943-957.
4. Raghu, G., et al., *Diagnosis of idiopathic pulmonary fibrosis. An official ATS/ERS/JRS/ALAT clinical practice guideline.* American journal of respiratory and critical care medicine, 2018. **198**(5): p. e44-e68.
5. Love, M.I., W. Huber, and S. Anders, *Moderated estimation of fold change and dispersion for RNA-seq data with DESeq2.* Genome biology, 2014. **15**(12): p. 1-21.
6. Subramanian, A., et al., *Gene set enrichment analysis: a knowledge-based approach for interpreting genome-wide expression profiles.* Proceedings of the National Academy of Sciences, 2005. **102**(43): p. 15545-15550.
7. Ertay, A., et al., *WDHD1 is essential for the survival of PTEN-inactive triple-negative breast cancer.* Cell Death & Disease, 2020. **11**(11): p. 1001.

Raw data for western blots

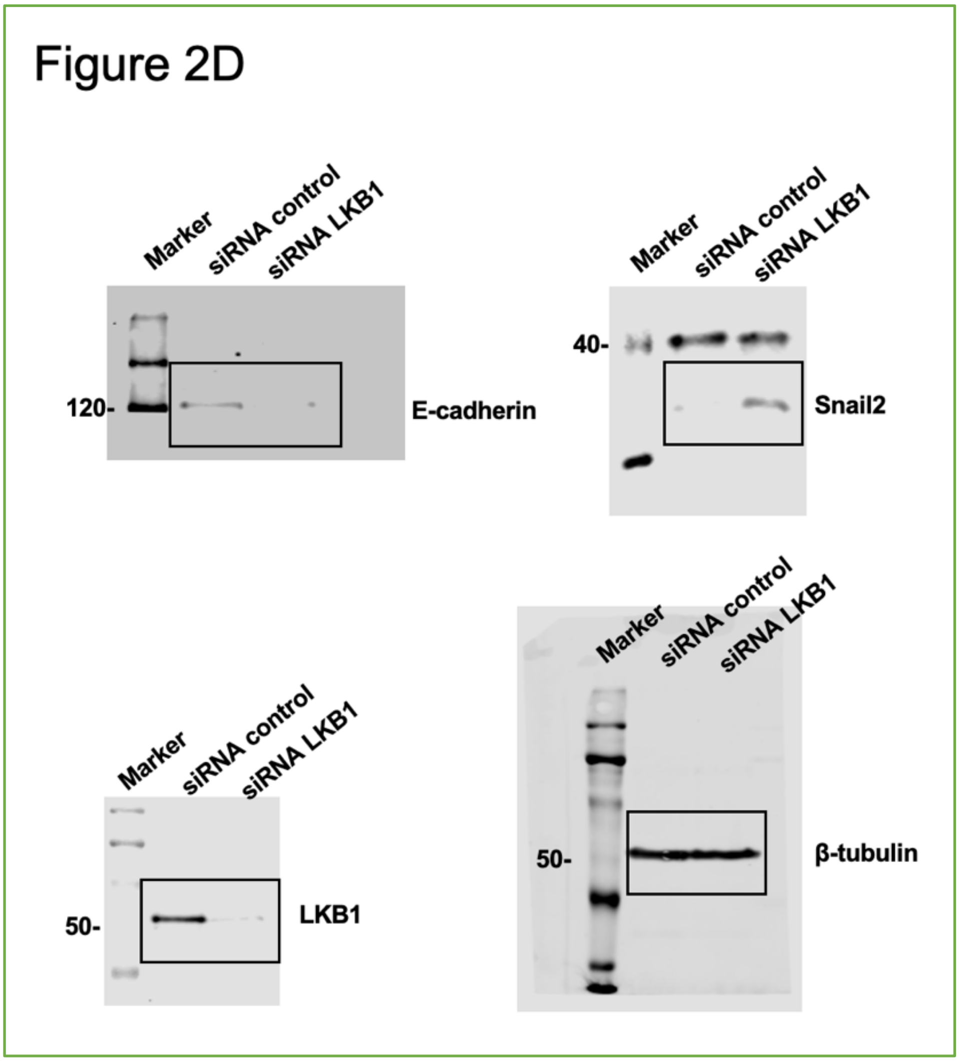

Figure 3A

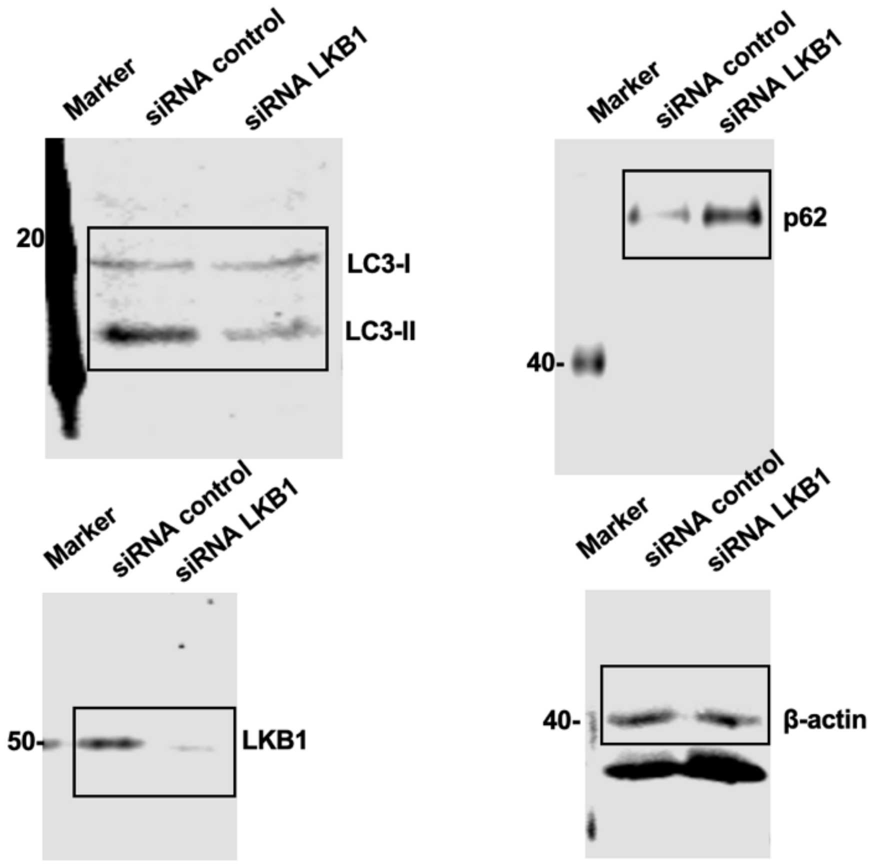
Marker
siRNA control
siRNA LKB1
20
LC3-I
LC3-II
Marker
siRNA control
siRNA LKB1
p62
40-
Marker
siRNA control
siRNA LKB1
50-
LKB1
Marker
siRNA control
siRNA LKB1
40-
β-actin

Figure 3D

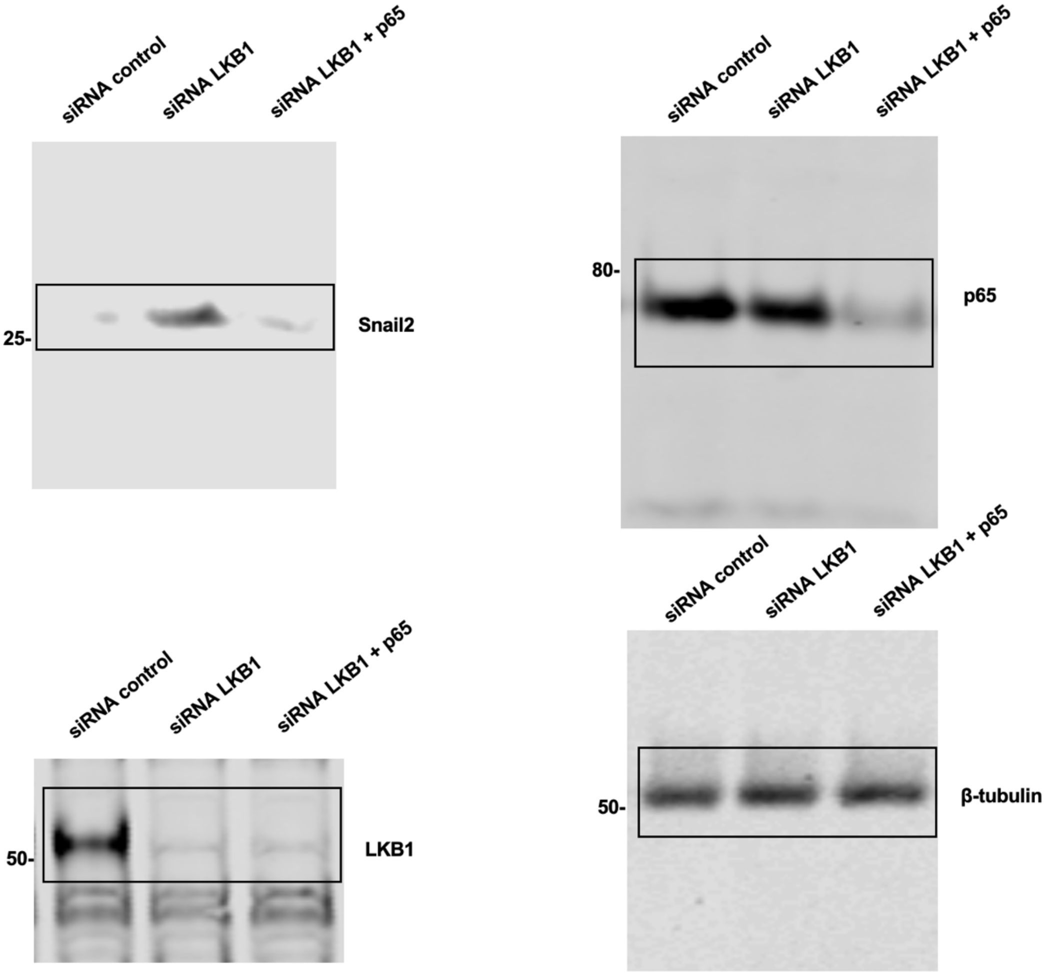
siRNA control
siRNA LKB1
siRNA LKB1 + p65
25-
Snail2
siRNA control
siRNA LKB1
siRNA LKB1 + p65
80-
p65
siRNA control
siRNA LKB1
siRNA LKB1 + p65
50-
LKB1
siRNA control
siRNA LKB1
siRNA LKB1 + p65
50-
β-tubulin

Figure 3E

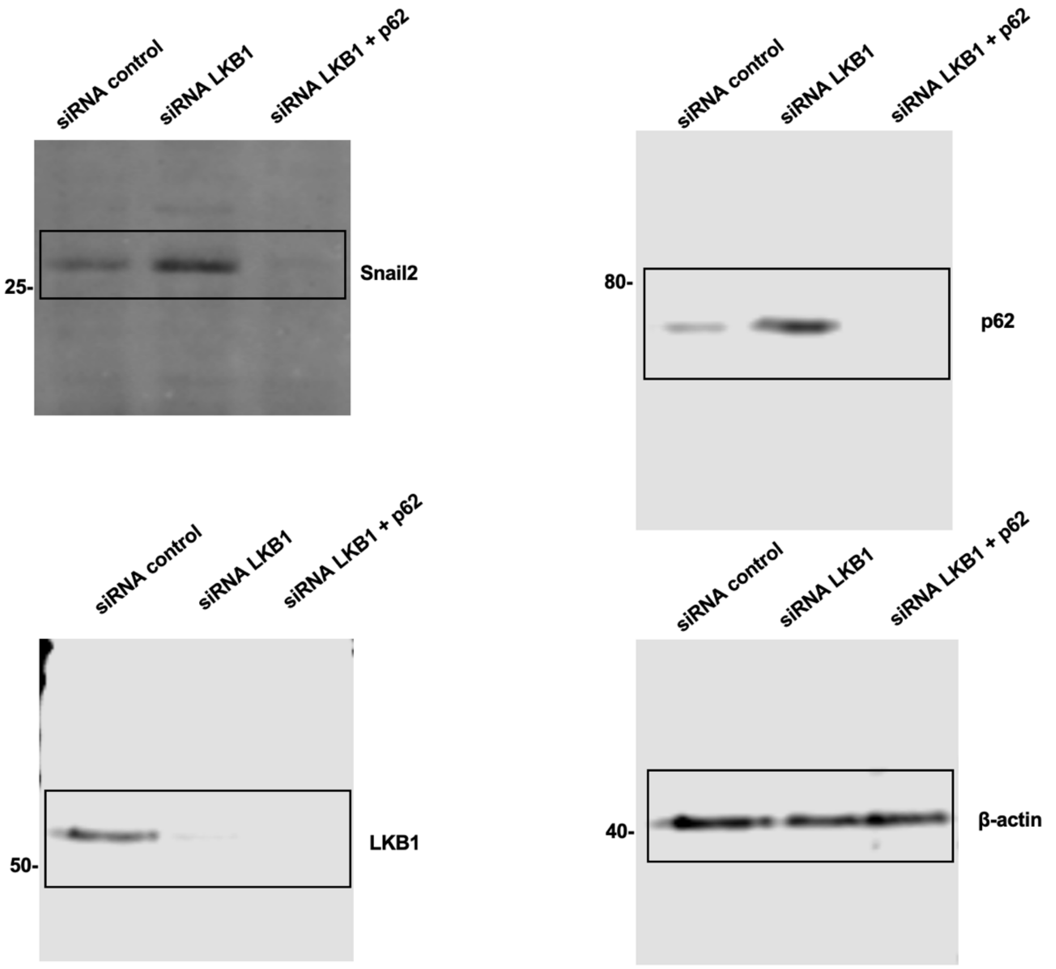
siRNA control
siRNA LKB1
siRNA LKB1 + p62
25-
Snail2
80-
p62
50-
LKB1
40-
β-actin

Figure 5F

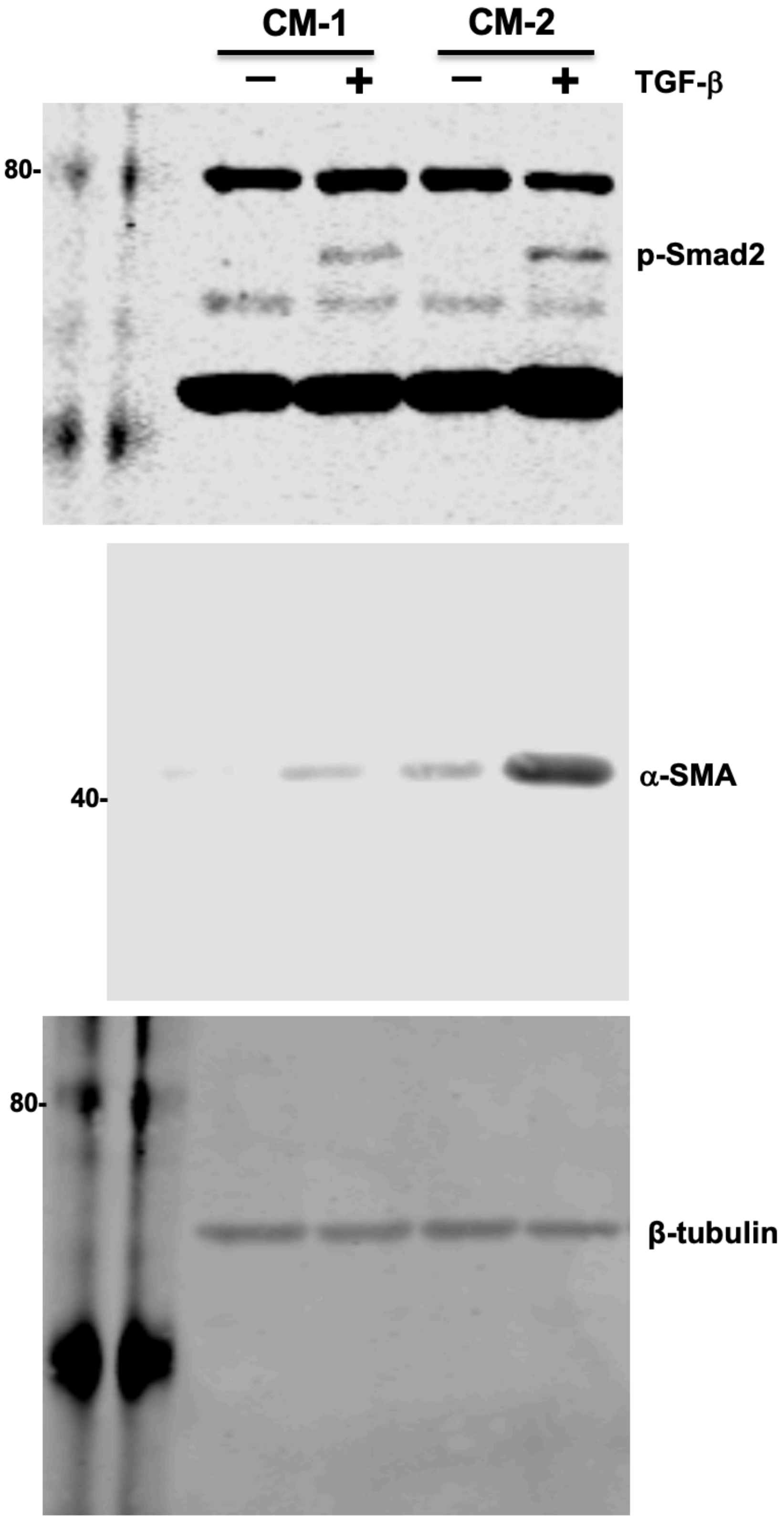

CM-1: control ATII CM
CM-2: LKB1-deplted ATII CM
